# Psychometric properties of the Arabic version of the 12-item diabetes fatalism scale

**DOI:** 10.1371/journal.pone.0190719

**Published:** 2018-01-11

**Authors:** Ola Sukkarieh-Haraty, Leonard E. Egede, Joelle Abi Kharma, Maya Bassil

**Affiliations:** 1 Alice Ramez Chagoury School of Nursing, Lebanese American University, Byblos, Lebanon; 2 Medical College of Wisconsin, Milwaukee, Wisconsin, United States of America; 3 Department of Natural Sciences, School of Arts and Sciences, Lebanese American University, Beirut, Lebanon; Public Library of Science, UNITED KINGDOM

## Abstract

**Background:**

There are widespread fatalistic beliefs in Arab countries, especially among individuals with diabetes. However, there is no tool to assess diabetes fatalism in this population. This study describes the processes used to create an Arabic version of the Diabetes Fatalism Scale (DFS) and examine its psychometric properties.

**Methods:**

A descriptive correlational design was used with a convenience sample of Lebanese adults (N = 274) with type 2 diabetes recruited from a major hospital in Beirut, Lebanon and by snowball sampling. The 12- item Diabetes Fatalism Scale- Arabic (12-item DFS-Ar) was back-translated from the original version, pilot tested on 22 adults with type 2 diabetes and then administered to 274 patients to assess the validity and reliability of the scale. Confirmatory factor analysis (CFA) was used to test the hypothesized factor structure. Cronbach’s alpha was used to test for reliability.

**Results:**

CFA supported the existence of the three factor hypothesis of the original DFS scale. The five items measuring “emotional distress” loaded under Factor 1, the four items measuring “spiritual coping” loaded under factor 2 and the last three items measuring “perceived self-efficacy” of the original scale loaded under Factor 3 (p <0.001 for all three subscales). Goodness of fit indices confirmed adequateness of the CFA model (CFI = 0.97, TLI = 0.96, RMSEA = 0.067 and pclose = 0.05). The 12-item DFS-Ar showed good reliability (Cronbach’s alpha of 0.86) and significantly predicted HbA1c (β = 0.20, p < 0.01). After adjusting for the demographic characteristics and the number of diabetes comorbid conditions, the 12-item DFS-Ar score was independently associated with HbA1c in a multivariable model (β = 0.16, p < 0.05).

**Conclusions:**

The 12-item DFS-Ar demonstrated good psychometric properties that are comparable to the original scale. It is a valid and reliable measure of diabetes fatalism. Further testing with larger and non-Lebanese Arabic population is needed.

## Introduction

Diabetes self-care is challenging and requires commitment and perseverance to adhere to medication regimen, blood glucose monitoring, healthy dietary choices, regular exercise and routine foot care [1]. It is important to understand how ethnic, cultural, religious, gender and socioeconomic factors influence diabetes control and outcomes [1]. Fundamental barriers to adherence may include patient factors (carrying out the required diabetes self-care regimen), system factors (inadequate social or medical support), psychosocial factors (fear, depression or social support) and health beliefs that are rooted in cultural values and conceptions [1]. Behavioral research in fatalism and its impact on health outcomes, particularly in chronic diseases, has surfaced in the past decades. The interest has been prompted by the fact that fatalistic beliefs are associated with poor intentions to change behavior resulting in a wide array of suboptimal health outcomes [2, 3]. In diabetes, one of the major health beliefs that have been documented as a hindering factor to adequate diabetes self-care, glycemic control and health outcomes is diabetes fatalism [4, 5]. Diabetes fatalism is defined as “a complex psychological cycle characterized by perceptions of despair, hopelessness, and powerlessness” [4, 5]. Diabetes fatalism has been negatively associated with poor medication adherence, diabetes self-care, uncontrolled glycemic levels, and decreased quality of life [4, 6, 7]. Fatalistic attitudes are also negatively correlated with optimal diabetes lifestyle (i.e., exercise, eating healthy foods and nonsmoking) [8]. Diabetes fatalism has been studied extensively in African American population [5], general United States population in the Southeast [5–7, 9–11], and British South Asians, particularly Muslims among them [12].

### Diabetes in Lebanon

The International Diabetes Federation (IDF) estimates that in the Middle East and the North African region, 1 in 11 adults has diabetes [13]. Being a developing country, diabetes prevalence in Lebanon is quite high, accounting for 8.5% [14], which is creating an economic burden on national health care expenditures. In Lebanon, patients with diabetes are more likely to endorse fatalistic attitudes to justify non-adherence to diabetes self-care [15]. In a cross sectional national survey conducted by Costanian and colleagues (2014), adherence to diabetes self-care and management was insufficient, which resulted in significantly higher prevalence of diabetes related complications, namely heart disease (27.8%) and retinopathy (16.6%) [14]. A recent cross-sectional study [15] revealed that diabetes self-care is strongly embedded in cultural values and beliefs. Despite uncontrolled glycemic levels, patients demonstrated low levels of diabetes-specific emotional distress. Possible explanations for this finding are denial of disease, hopelessness, or poor self-efficacy regarding self-care [15]. These explanations are consistent with the concept of diabetes fatalism. To our knowledge, there is no valid or reliable measure of diabetes fatalism in Arabic or in Lebanese patients. As a result, it is imperative to develop a valid and reliable measure of diabetes fatalism in this population. This study describes the processes used to create an Arabic version of a previously validated Diabetes Fatalism Scale [4] and examine its psychometric properties in a Lebanese population with type 2 diabetes.

## Methods

### Participants

A descriptive correlational design was used with a convenience sample of Lebanese adults (N = 274) recruited from a medical center in Beirut, Lebanon and by snowball sampling defined as “a chain-referral method where initial participants (seeds) recruit others from their social network” [16]. Inclusion criteria included patients diagnosed with type 2 diabetes for more than one year, on oral hypoglycemic pills, no psychiatric illness, and ability to read and write Arabic. Physicians screened patients using the inclusion criteria and referred those eligible for the study. Interested participants were approached by the researcher, informed of the study, and asked to fill out study questionnaire after providing their written informed consent. Additionally, the researcher obtained demographic data (age, gender, education, marital status, occupation and income), health information (diabetes type, duration of disease, family history, smoking status, presence of diabetes related complications, height and weight) and most recent glycated hemoglobin (HbA1c). The study received the approval of the Institutional Review Board of the Lebanese American University and Lebanese American University Medical Center, and has been performed in accordance with the ethical standards as laid down in the 1964 Declaration of Helsinki and its later amendments.

### Diabetes Fatalism Scale-12 (DFS)

Diabetes fatalism is operationally defined as ‘‘a complex psychological cycle characterized by perceptions of despair, hopelessness, and powerlessness” [4]. DFS is the only published well-validated scale that measures diabetes fatalism. It consists of 12 items conceptualized in three subscales: 1) emotional distress (despair); 2) religious and spiritual coping (hopelessness); and 3) perceived self-efficacy (powerlessness). Items are scored on a 6-point Likert scale with scores ranging from 1 = strongly disagree to 6 = strongly agree. Higher scores represent more fatalistic attitudes towards diabetes. The DFS-12 is a well-established tool with internal consistency of a Cronbach's alpha = 0.804 and has been shown to be independently associated with increased HbA1c. It has been extensively examined in the general US population [4–7, 9–11].

### Translation process and pilot test

Permission to translate the English tool was obtained from the author of the original scale [4]. Back-translation method was implemented to create the Arabic version [17]. The 12-item DFS-Ar instrument underwent two phases of translation. In the first phase, forward translation, two bilingual researchers, whose native language is Arabic, translated the instrument from English to Arabic. Then, a bilingual, certified translator translated the Arabic version back to English, creating the backward translation. A panel of three experts in diabetes compared the final back translated version to the original English version for content validity and cultural suitability. In the final phase, content and conceptual equivalence were achieved after a pilot test was performed with 22 patients with type 2 diabetes from a diabetes outpatient clinic who had the same selection criteria as the study sample. The questionnaire was well received during the pilot test and no further changes were deemed necessary. Cronbach’s alpha of the translated scale revealed comparable results to the original instrument (α = 0.812) at pilot testing. Corrected total-item correlation of the 12 item scale ranged from—0.39 to 0.769.

### Statistical analyses

Analysis was performed using STATA v14. Categorical variables were described using frequencies and percentages, while means and standard deviations were used to represent continuous data. A Confirmatory Factor Analysis (CFA) was performed to answer the question whether the theoretical 3 factor diabetes fatalism model proposed by the original author may be confirmed using structural equation modeling in a Lebanese adult population. Standard CFA models were used that is every item loads on only one factor with no correlations between measurement errors. The model parameters were estimated using maximum likelihood estimation. Three goodness-of-fit indices were used to evaluate the model's fit: the Comparative Fit Index (CFI) and the Tucker Lewis Index (TLI) with > 0.90 and > 0.95 for acceptable and excellent fit respectively; and the Root Mean Square Error of Approximation (RMSEA) with < 0.80 for reasonable fit; along with its corresponding PCLOSE; best if above 0.05. Reliability statistics for items loading on each factor alone was performed using Cronbach’s alpha, corrected item-total correlations, and Cronbach’s alpha without the item. A simple linear regression was used to assess how much of the variance in HbA1c was explained by 12-item DFS-Ar. A multiple linear regression was also used to assess the independent effect of 12-item DFS-Ar on HbA1c after controlling for demographic characteristics, diabetes duration and number comorbid conditions.

## Results

### Demographic and physiologic characteristics of the participants

Demographic and physiologic characteristics are presented in Table 1. The mean age of the participants was 58.2 (SD = 3.4) and the majority were females (53.7%) The mean HbA1c in the sample was 7.9 (SD = 1.9), while only 10.7% of the participants reported having no diabetes-related complications (Table 1).

**Table 1:** Demographic characteristics of study participants*.

	*M (SD)*
Age	58.24 (13.48)
Hemoglobin A1c	7.90 (1.90)
	N (%)
**Gender**	
Female	53.76%
**Social status**	
Single	18 (6.43%)
Married	208 (74.29%)
Divorced	15 (5.36%)
Widowed	39 (13.93%)
**Level of education**	
< 11 years	110 (39.57%)
High school	76 (27.34%)
Technical	25 (8.99%)
Undergraduate degree	40 (14.39%)
Graduate degree	27 (9.71%)
**Occupation**	
Employed	119 (43.91%)
Unemployed	139 (51.29%)
Unable to work due to health condition	13 (4.80%)
**Insurance**	
Yes	165 (59.35%)
**Income**	
Have more than enough to make ends meet	55 (20.52%)
Have enough to make ends meet	147 (54.85%)
Do not have enough to make ends meet	66 (24.63%)
**Diabetes duration**	
< 5 years	90 (32.37%)
5–10 years	97 (34.89%)
At least 11 years	91 (32.73%)
**Family history**	
Yes	205 (73.74%)
**Number of comorbid conditions**	
0	30 (10.75%)
1	94 (33.69%)
2	69 (24.73%)
3+	86 (30.82%)

* Data is presented as mean ± SD for continuous variables and N (%) for categorical variables

### Confirmatory factor analysis

**Fig 1:**
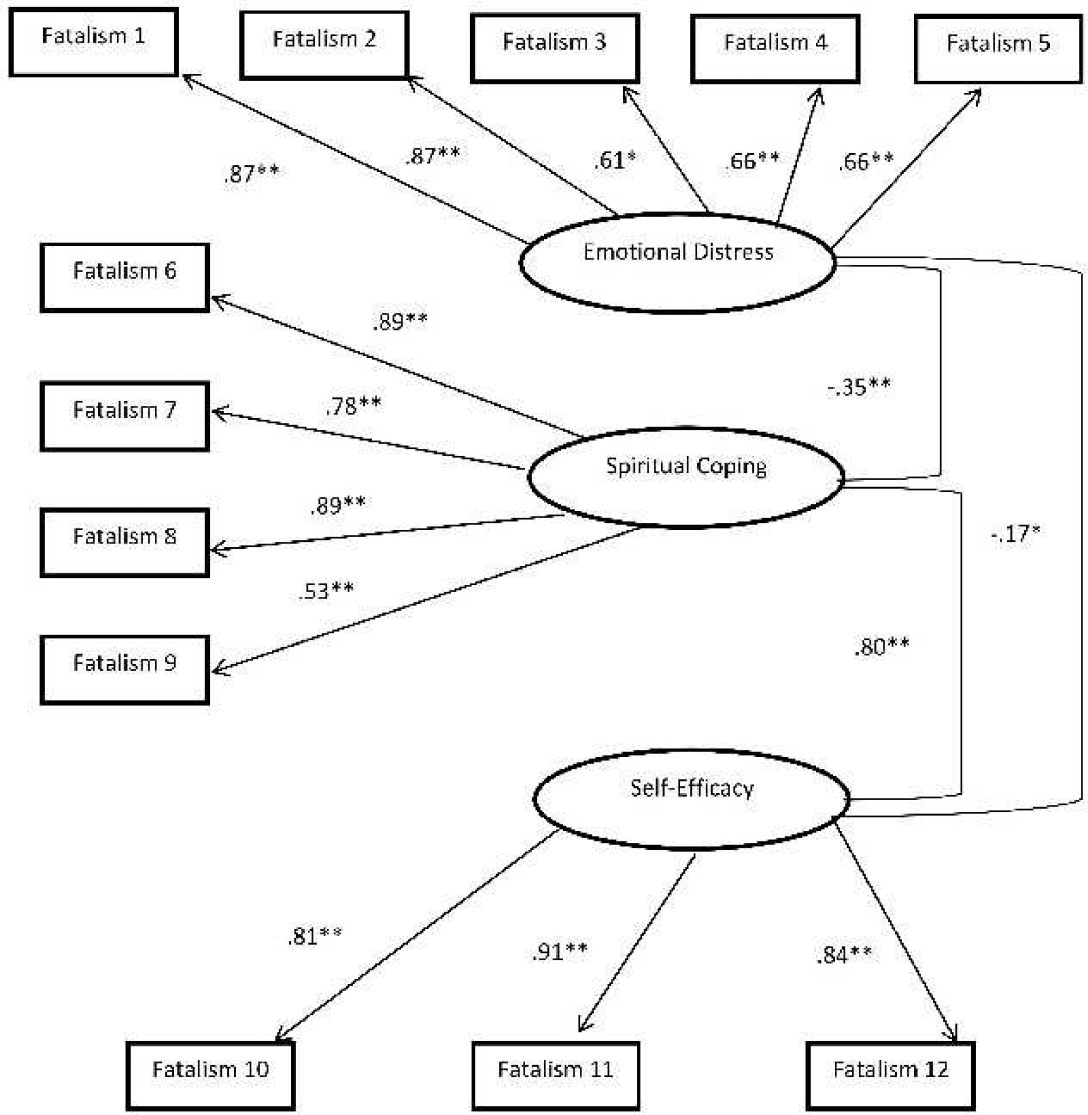
Structural equation model with standardized path coefficients of Diabetes Fatalism Scale dimensions. The fit of the final model was as follows: R2 = 99, root mean square error of approximation = 0.067, pclose = 0.05, comparative fit index = 0.97. *p < 0.05. **p < 0.01.

A primary CFA model was hypothesized to be comprised of three factors: emotional distress, spiritual coping and perceived self-efficacy corresponding to the original DFS-12 subscales. Items 1 through 5 were hypothesized to load on the emotional distress factor while items 6 through 9 and 10 through 12 were hypothesized to load on spiritual coping and perceived self-efficacy respectively. Results of factor loading coefficients revealed that the hypothesized 3- factor model fit the data. The factor loading ranged from 0.61 to 0.86 for the emotional distress subscale (p< 0.001), from 0.52 to 0.89 for the spiritual coping subscale (p <0.001) and from 0.81 to 0.90 for the perceived self-efficacy (p < 0.001). Goodness of fit indices also confirmed adequateness of the model with CFI = 0.97, TLI = 0.96 and RMSEA = 0.067 with PCLOSE of 0.05 (Fig 1).

The correlations among the 3 factors in the hypothesized confirmatory model were also calculated. The "emotional distress" correlated negatively with the "spiritual coping" and the "self-efficacy" subscales (r = -0.35, p< 0.001 and r = -.17, p < 0.05). The "spiritual coping" correlated positively with the "perceived self-efficacy" subscales (r = 0.80, p < 0.001). These were consistent with the original DFS-12 findings [4].

### Internal consistency and reliability

The 12-item DFS-Ar analysis revealed a Cronbach’s alpha of 0.86. The item analyses of the three subscales revealed Cronbach’s alphas of 0.87 for subscale 1(emotional distress), 0.85 for subscale 2 (spiritual coping) and 0.89 for subscale 3 (perceived self-efficacy) respectively. Corrected item-total correlation ranged from 0.64 to 0.79 for subscale 1, from 0.51 to 0.81 for subscale 2, and from 0.75 to 0.81 for subscale 3. The item analysis revealed that dropping any one item from the scale did not improve Cronbach’s alpha significantly. Table 2 presents the item, scale, subscale means with standard deviations.

**Table 2:** Descriptive statistics of the diabetes fatalism scale*.

	M (SD)	Corrected item-total correlation	Cronbach’s α without item
**Subscale 1 Emotional Distress (α = 0.87)**	17.21 (7.09)		
1. I get upset when I think about my diabetes	3.50 (1.82)	0.73	0.83
2. I feel down when I think about my diabetes	3.46 (1.78)	0.79	0.82
3. I get frustrated with having to live with diabetes	3.35 (1.78)	0.64	0.86
4. Diabetes is a disease that makes life more difficult	3.56 (1.68)	0.65	0.85
5. Diabetes causes a lot of suffering for me	3.34 (1.67)	0.65	0.85
**Subscale 2: Religious and spiritual coping (α = 0.85)**	13.3 (6.40)		
6. Trusting in God has helped me better deal with my diabetes	3.27 (1.97)	.76	0.79
7. I believe God does not give me more than I can bear	3.20 (1.92)	.71	0.81
8. I believe God can completely cure my diabetes	3.21 (1.97)	.81	0.76
9. I have prayed about my diabetes so I am not going to worry about it anymore	3.62 (1.78)	.51	0.89
**Subscale 3: Perceived Self-efficacy (α = 0.89)**	9.63 (4.99)		
10. I believe I am able to control my diabetes the way my doctor expects	3.25 (1.72)	0.75	0.87
11. If I do everything my doctor tells me, I can prevent the complications of diabetes like blindness, amputations, kidney failure, impotence, etc.	3.19 (1.86)	0.81	0.82
12. I believe that diabetes is controllable **12-item Diabetes Fatalism Scale (α = 0.86)**	3.19 (1.83) 40.14 (11.35)	0.78	0.85

* Items 6 through 12 are reverse scored (1 = 6) (2 = 5) (3 = 4) (4 = 3) (5 = 2) (6 = 1)

### Relationship of 12-item DFS-Ar with HbA1c

The 12-item DFS-Ar was significantly associated with mean HbA1c in a univariate linear regression model (β = 0.21, p < 0.01). The scale explained 4% of the variance in HbA1c. After controlling for demographic characteristics, diabetes duration and number of comorbid conditions, the 12-item-12-item DFS-Ar score was still independently associated with HbA1c (β = 0.16, p < 0.05) (Table 3). The combination of the 12-item DFS-Ar score, demographic characteristics and number of comorbid conditions explained 10.3% of the variance in HbA1c in the sample.

**Table 3:** Independent relationship between 12-item DFS and demographic characteristics and hemoglobin A1c.

Variables	Coefficient	Beta†	P-value
*DFS*	0.03	0.16*	0.015
*Age (Years)*	-0.02	-0.16	0.057
*Gender* ^a^	-0.70	-0.18*	0.014
*Social Status* ^b^			
Married	0.19	0.04	0.711
Divorced	-0.27	0.03	0.726
Widowed	0.33	0.06	0.595
*Level of Education* ^c^			
High School	0.39	0.09	0.221
Technical	0.60	0.09	0.170
Undergraduate Degree	0.34	0.06	0.389
Graduate Degree	1.00	0.16*	0.045
*Occupation* ^d^			
Unemployed	0.50	0.13	0.113
Unable to work due to health condition	0.14	0.02	0.805
*Income* ^e^			
Have enough to make ends meet	-0.29	-0.08	0.382
Do not have enough to make ends meet	-0.40	-0.09	0.309
*Insurance* ^f^	0.11	0.03	0.668
*Diabetes duration* ^g^			
5–10 years	0.33	0.08	0.276
At least 11 years	0.58	0.14	0.082
*Number of comorbid conditions* ^h^			
1	-0.13	-0.03	0.757
2	-0.11	-0.02	0.810
3+	1.16	0.28*	0.009

^a^ reference group “male”

^b^ reference group “single”

^c^ reference group “< 11 years”

^d^ reference group “employed”

^e^ reference group “have more than enough to make ends meet”

^f^ reference group “yes”

^g^ reference group “< 5 years”

^h^ reference group “0”.

* *p* < 0.05.

†Beta–standardized beta coefficient

## Discussion

This study provides initial evidence of the psychometric properties of the 12-item DFS-Ar. We found that the translated version is a valid and reliable instrument that assesses fatalism in patients with type 2 diabetes. Overall, the Arabic version performed fairly well and the results were comparable to the English version [4]. The 12-item DFS-Ar was significantly correlated with glycemic control, and a set of demographic characteristics.

The reliability of the 12-item DFS-Ar revealed high internal consistency of the overall scale (α = 0.86) as well as the subscales (0.87 for emotional distress, 0.85 for spiritual coping and 0.89 for perceived self-efficacy). The reliability was further confirmed since removing one item from the scale did not improve significantly the internal consistency of the overall scale. Our findings were consistent and comparable with the reliability analyses of the original 12-item DFS [4].

The review process conducted by the expert panel confirmed the conceptual content of the Arabic version whereby all items were translated appropriately. The construct validity of the 12-item DFS-Ar is supported by two results. First, we used CFA to evaluate the theoretical models of 12-item DFS with three factors and to test whether the theory is supported by data. Results of CFA show that the data supports the theoretical model of diabetes fatalism in concordance with the original 12-item DFS [4]. The assessment of goodness of fit in a CFA study usually involves one factor structure and one group for construct validity evaluation, one factor structure but multiple groups for response pattern comparison, and different factor structures for competing model comparison [18]. Hence, our results of goodness of fit is reasonable. The overall content structure is remarkably clear and indicates a good factorial validity. The results from the current study suggest that the Arabic version of 12-item DFS demonstrated sound psychometric properties to assess diabetes fatalism among Arabic speaking people with diabetes for research or clinical use.

Second, 12-item DFS-Ar was independently associated with HbA1c in a univariate linear regression model (β = 0.21, p < 0.01), whereby 12-item DFS-Ar scores explained 4% of the variance in HbA1c in the sample. Our results are commensurate with the 12-item DFS whereby scores alone explained 4% of the variance in HbA1c in the sample (beta = 0.20, p = 0.004) [4]. The combination of the 12-item DFS-Ar score, demographic characteristics and number of comorbid conditions explained 10.3% of the variance in HbA1c in the sample, which is similar to what was found in the original 12-item DFS where the multivariable model explained 30% of the variance in HbA1c [4]. Additionally, the results further confirms that the three constructs of the 12-item DFS-Ar (emotional distress, coping, and self-efficacy) are theoretically sound and have been shown to correlate with glycemic control.

The results of this study need to be interpreted with respect to design and sampling limitations. The study provides preliminary analyses of psychometric properties of the 12-item DFS-Ar. However, additional work is needed to evaluate test-retest reliability. Second, we had a convenience sample and participants were predominantly of lower socio-economic status and on oral hypoglycemic pills. Therefore, further validation in a more diverse sample is warranted. Finally, the 12-item DFS-Ar was developed and tested among Lebanese patients with type 2 diabetes. Although fatalistic attitudes are common among Arabs [19], further testing is diverse Arabic speaking populations will be necessary to establish cross-cultural validity. Additionally, we did not use other measures of theoretically-related constructs to measure the convergent validity of 12-item DFS-Ar. This approach will serve as the basis for future research directions.

In conclusion, this study describes the processes used to create an Arabic version of the Diabetes Fatalism Scale and examine its psychometric properties. This study provides initial evidence of the psychometric properties of the 12-item DFS-Ar. We found that the translated version is a valid and reliable instrument that assesses fatalism in patients with type 2 diabetes. Overall, the Arabic version performed fairly well and the results were comparable to the English version [4]. The 12-item DFS-Ar was significantly correlated with glycemic control, and a set of demographic characteristics. It may serve multiple purposes in clinical settings and research studies to assess patients’ levels of emotional despair, hopelessness, and powerlessness associated with diabetes.

The data set is available in S1 File.

## Supporting information

S1 File Dataset of the study variables. (XLS)
